# RNA thermometers are widespread upstream of ABC transporter genes in bacteria

**DOI:** 10.1016/j.jbc.2024.107547

**Published:** 2024-07-09

**Authors:** Alina Y. Tong, Elisha L. Tong, Michael A. Hannani, Samantha N. Shaffer, Danna Santiago, Adrian R. Ferré-D’Amaré, Luiz F.M. Passalacqua, Michael M. Abdelsayed

**Affiliations:** 1 Department of Biology, California Lutheran University, Thousand Oaks, California, USA; 2 Laboratory of Nucleic Acids, National Heart, Lung, and Blood Institute, National Institutes of Health, Bethesda, Maryland, USA

**Keywords:** RNA, ABC transporter, X-ray crystallography, RNA structure, gene regulation, translation regulation, noncoding RNA, heat stress

## Abstract

RNA thermometers are temperature-sensing non-coding RNAs that regulate the expression of downstream genes. A well-characterized RNA thermometer motif discovered in bacteria is the ROSE-like element (repression of heat shock gene expression). ATP-binding cassette (ABC) transporters are a superfamily of transmembrane proteins that harness ATP hydrolysis to facilitate the export and import of substrates across cellular membranes. Through structure-guided bioinformatics, we discovered that ROSE-like RNA thermometers are widespread upstream of ABC transporter genes in bacteria. X-ray crystallography, biochemistry, and cellular assays indicate that these RNA thermometers are functional regulatory elements. This study expands the known biological role of RNA thermometers to these key membrane transporters.

ATP-binding cassette (ABC) transporters are a superfamily of transmembrane proteins that harness ATP hydrolysis to facilitate the export and import across cellular membranes of an extensive array of substrates, such as metal ions, sugars, amino acids, peptides, iron chelators, vitamins, and drugs (1, 2, 3). These transmembrane transporters feature a structural arrangement comprising at least four domains: two transmembrane domains (TMDs) and two ATP-binding domains (ABC domains) (2, 4). The ABC domains of these transporters exhibit a high degree of sequence conservation (2, 5). In contrast, the TMDs are highly variable and specific for the substrate of the transporter, leading to a wide array of transporter types (2, 5). In prokaryotes, some ABC transporters have an additional fifth domain that contains a high-affinity binding protein for the substrate being transported (2, 5).

In bacteria, ABC transporters are instrumental in responding to environmental conditions such as osmotic and oxidative stress, contributing to the survival of bacteria in the host environment (3, 4). Additionally, ABC transporters are implicated in critical biological processes such as virulence and pathogenesis through nutrient acquisition and multidrug resistance (6, 7). Although response to heat shock is critical to all cellular processes, there is limited understanding of the interplay between heat stress and ABC transporters.

RNA thermometers are non-coding RNAs found in the 5′ untranslated regions (5′-UTRs) of genes, where they regulate gene expression in response to changes in temperature (8, 9). A well-characterized RNA thermometer motif discovered in bacteria is the ROSE-like element (repression of heat shock gene expression). ROSE-like thermometers contain a conserved U(U/C)GCU motif that imperfectly base pairs with the Shine-Dalgarno (SD) sequence in a stem with a predicted bulged G nucleotide (8, 10, 11). Increases in temperature promote destabilization of the motif followed by exposure of the SD for ribosomal binding, resulting in increased gene expression (8, 10, 11). ROSE-like RNA thermometers are present upstream of several heat shock and virulence-associated genes in bacteria (12).

We used bioinformatics to discover the prevalence of ROSE-like RNA thermometers in bacteria. Manual curation of our search revealed an abundance of potential ROSE-like RNA thermometers upstream of different ABC transporter genes. Previously, two ROSE-like RNA thermometers were predicted to lie upstream of ABC transporter genes (13), and a non-ROSE-like RNA thermometer was validated in *Yersinia pseudotuberculosis* upstream of *oppA* (14). *oppA* is part of an oligopeptide permease ABC transport operon (opp), consisting of five genes (*oppA*, *oppB*, *oppC*, *oppD*, and *oppF*) that make up the oligopeptide transport system (15, 16). *oppA* is responsible for oligopeptide binding and is necessary for oligopeptide uptake by the Opp system (16). Interestingly, one of the candidate sequences we focused on in this study is upstream of another component of the opp operon, *oppF*. The *oppF* gene encodes an ATP-binding protein, which is one of the ABC domain components of the oligopeptide ABC transporter and is part of the opp operon (16). The opp operon is essential for the uptake of nutrients and virulence in pathogenic bacteria (17). In this study, we sought to elucidate the prevalence and the molecular mechanism governing the temperature-dependent expression of diverse ABC transporter genes across the bacterial kingdom.

## Results

We identified potential RNA thermometer sequences by utilizing Robo-Therm, a bioinformatics-based pipeline to discover RNA thermometers (18). Robo-Therm utilizes the RNA motif search tool RNArobo (19), which allows users to fully handcraft and feature components of an RNA that are essential for its function. We recently used Robo-Therm to discover several fourU thermometers including an RNA thermometer upstream of the *blyA* gene that occurs in the genomes of the SPβ prophage and its host, *Bacillus subtilis* 168 (20), a thermometer upstream of the gene *tetR* that occurs in the genomes of *Escherichia coli* and *Shigella flexneri*, and a thermometer upstream of the gene *σ*^*70*^ that is found in the genomes of *Mediterraneibacter gnavus*, *Bacteroides pectinophilus*, and the bacteriophage *Caudoviricetes* (18).

From our bioinformatic predictions (19), we identified potential ROSE-like RNA thermometers by generating a search template based on the secondary structure of a previously described ROSE-like thermometer upstream of the *ibpA* gene in various *Pseudomonas* species (11). The *ibpA* thermometers display the characteristic ROSE-like U(U/C)GCU motif with a predicted bulged G nucleotide. Our search revealed 41 potential ROSE-like RNA thermometer sequences upstream of different ABC transporter genes, including genes encoding the TMDs, ABC domains, and the additional high-affinity substrate binding protein domain (Table S1). The alignment of our candidates reveals the conservation of the putative ROSE-like motif UUGCU.

**Figure 1:**
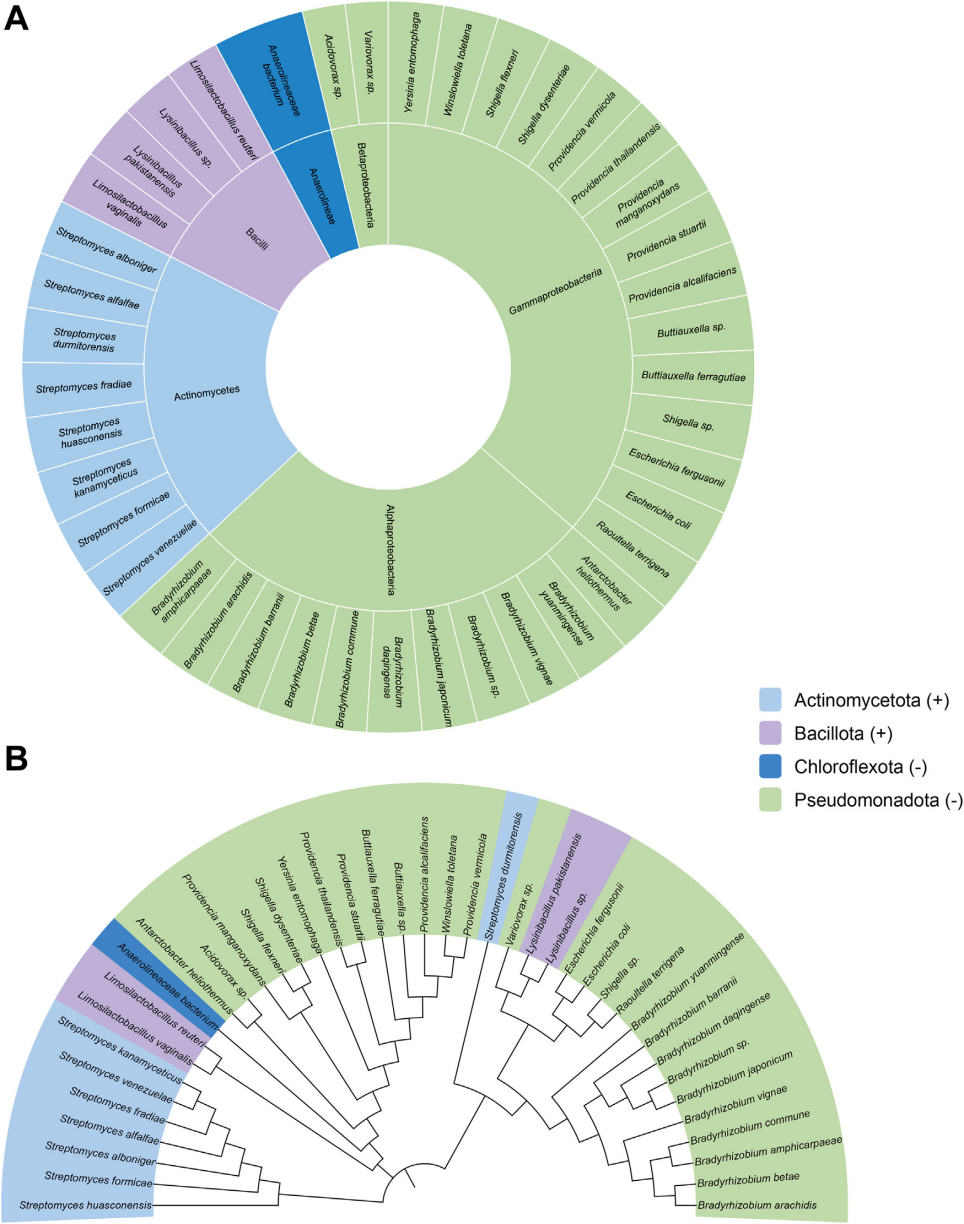
**Widespread occurrence of ROSE-like RNA thermometers upstream of ABC transporter genes in bacteria.***A*, sunburst species distribution of ROSE-like RNA thermometer presence upstream of ABC transporter genes in bacteria. Inside tier of sunburst shows class distribution. *B*, phylogenetic tree representing the sequence relationship of 41 bacterial species that contain a ROSE-like RNA thermometer sequence upstream of ABC transporter genes. (*A*) and (*B*) are color-coded according to phyla. Gram-negative phyla are noted with a minus symbol and gram-positive phyla with a positive symbol.

These RNA sequences were found in a diverse range of gram-positive and gram-negative bacteria. These classes include actinomycetes, alphaproteobacteria, bacilli, betaproteobacteria, and gammaproteobacteria, demonstrating the widespread occurrence of these RNA thermometers upstream of ABC transporter genes in bacteria (Fig. 1, *A* and *B*). Several highly virulent and multidrug-resistant pathogens are present in our results, including many bacteria that are found in the human gut microbiome (21). These thermometers are widespread among a variety of bacteria and are located upstream of different ABC transporter genes, indicating that RNA thermometers are highly conserved across bacterial ABC transporters.

**Figure 2:**
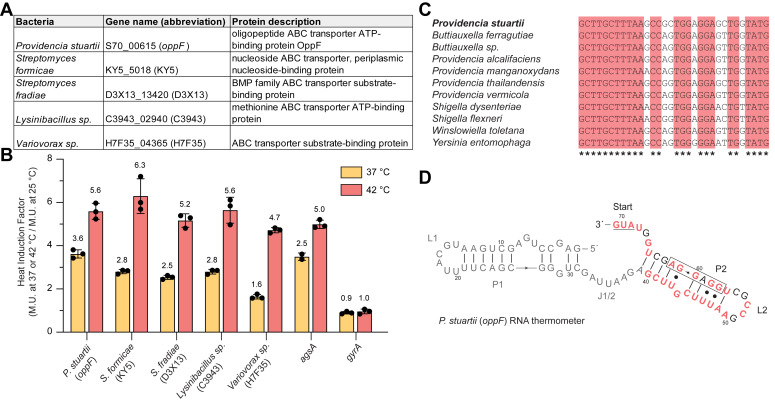
**RNA thermometer candidates upstream of ABC transporter genes.***A*, gene information of the five RNA thermometer candidates tested. *B*, heat induction factor of all five candidates tested. Heat induction factor [activity in Miller Units (M.U.) at 37 °C/25 °C or 42 °C/25 °C] indicated on top of each bar. The *agsA* RNA thermometer is a positive control (31), and DNA gyrase (*gyrA*) is a negative control (mean ± standard deviation; n = 3 biological replicates). For all five candidates, translation was significantly greater at 37 and 42 °C than at 25 °C and ranged from ∗∗∗ (*p* < 0.001) to ∗∗∗∗ (*p* < 0.0001); Student’s two-tailed *t* test. For individual *p* values, refer to Fig. S1. *C*, sequence alignment of 11 examples of ROSE-like RNA thermometers found upstream of the *oppF* gene. The confirmed RNA thermometer is in bold. For a complete list of sequences, refer to Table S1. *D*, predicted secondary structure of the tested ROSE-like RNA thermometer found upstream of the *oppF* gene in *P. stuartii*. Nucleotides conserved from (*C*) are in *red*. The Shine-Dalgarno sequence is boxed and AUG start codon is underlined.

We chose to focus on five candidate sequences that are representative of different phyla and also are upstream of different types of ABC transporter genes (Table 1). To determine the thermoregulatory activity of five candidate sequences, we used a reporter system containing the 5′-UTR of candidate genes upstream of a heat-stable β-galactosidase (*bgaB*) (Fig. S1*A*). Heat induction was tested in *E. coli* expressing the 5′-UTR-*bgaB* fusions at 25, 37, or 42 °C. β-galactosidase activity was measured for each temperature, and heat induction profiles were calculated for each RNA thermometer [activity in Miller Units (M.U.) at 37 °C/25 °C or 42 °C/25 °C]. *bgaB* fusions containing the extensively characterized *agsA* RNA thermometer were used as a positive control (22), and the 5′-UTR of the DNA gyrase gene (*gyrA*), which is not thermally regulated, was tested as a negative control. We verified five of our predicted RNA thermometers from our searches that occur in different ABC transporter genes in the genomes of *Providencia stuartii, Streptomyces formicae, Streptomyces fradiae, Lysinibacillus* sp., *Variovorax* sp., and altogether they exhibited a heat induction profile between ∼4.7- and 6.3-fold at 42 °C (Figs. 2, *A* and *B*, S1*B*).

**Table 1:** Genome information

Bacteria	Gene	Accession Number
*Providencia stuartii* MRSN 2154	*S70_00615*	CP003488.1
*Streptomyces formicae* strain KY5	*KY5_5018*	CP022685.1
*Streptomyces fradiae* strain NKZ-259 chromosome	*D3X13_13420*	CP032266.1
*Lysinibacillus* sp. B2A1 chromosome	*C3943_02940*	CP027224.1
*Variovorax* sp. PAMC26660 chromosome	*H7F35_04365*	CP060295.1

From our validated RNA thermometers, we focused on the *oppF* RNA thermometer in *P. stuartii* given that 11 potential RNA thermometers in our search are upstream of the same *oppF* gene (Table S1). Alignment of these sequences reveals a high degree of conservation, and all the identified *oppF* thermometer candidates include the ROSE-like motif (Fig. 2, *C* and *D*). We additionally chose to focus on the *oppF* thermometer given that the only other previously validated RNA thermometer associated with ABC transporters was discovered upstream of *oppA*, which is a component of the same operon. Heat shock of cells expressing *P. stuartii oppF-bgaB* fusions resulted in heat induction factors of ∼3.6- and 5.6-fold at 37 and 42 °C, respectively (Figs. 2*B* and S1*B*), demonstrating that the 5′-UTR of *P. stuartii oppF* modulates reporter gene activity in a temperature-dependent manner.

**Figure 3:**
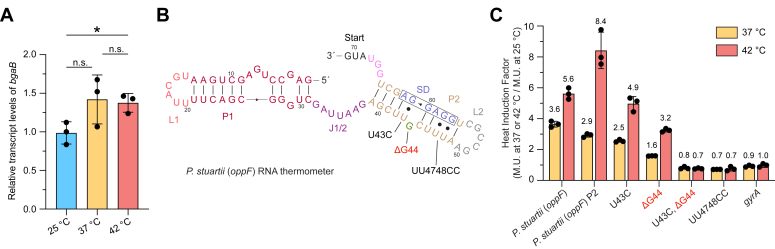
**Biochemical investigations of the *oppF* ROSE-like RNA thermometer from *P. stuartii*.***A*, relative transcript levels of the 5′-UTR of *oppF* at 25, 37, and 42 °C measured by qRT-PCR. Transcript levels were normalized to reference gene *gyrA* (mean ± standard deviation; n = 3 biological replicates, each with three technical replicates). No significant difference (n.s) exists between relative levels of the 5′-UTR of *oppF* transcripts at 37 °C compared to 25 °C and at 42 °C compared to 37 °C (*p* > 0.05). ∗ (*p* < 0.05) between relative levels of the 5′-UTR of *oppF* transcripts at 42 °C compared to 25 °C. Student’s two-tailed *t* test. *B*, predicted secondary structure depicting mutations tested for thermoregulation activity. Shine-Dalgarno sequence is boxed. *C*, heat induction factor of wild-type *oppF*, *oppF* P2, and *oppF* mutants, and DNA gyrase (*gyrA*) negative control (mean ± standard deviation; n = 3 biological replicates). Heat induction factor [activity in Miller Units (M.U.) at 37 °C/25 °C or 42 °C/25 °C] indicated on top of each bar. Translation of the *oppF* wild-type UTR was significantly different from translation of *oppF* mutants at 42 °C and ranged from ∗ (*p* < 0.05) to ∗∗∗∗ (*p* < 0.0001); Student’s two-tailed *t* test. For individual *p* values, refer to Fig. S2.

To distinguish between transcriptional and translational control in the system, transcript levels of *P. stuartii oppF-bgaB* fusions were measured by quantitative real-time PCR (qRT-PCR). Cells were harvested under the same conditions of β-galactosidase assays at 25, 37, and 42 °C. Heat induction resulted in a modest ∼1.4-fold increase in transcript abundance at 37 and 42 °C (Fig. 3*A*). Additionally, there was no increase in transcript abundance between 37 and 42 °C, contrasting the almost 2-fold difference of β-galactosidase activity at 37 and 42 °C (Fig. 2*B*), indicating that the majority of regulation is achieved at the post-transcriptional level.

To further validate that the *P. stuartii oppF* RNA thermometer sequence is directly responsible for increased heat induction, mutations were made to strengthen and stabilize the base pairing of the ROSE-like–containing stem (Figs. 3, *B* and *C* and S2). The U43C and UU4748CC stabilizing mutations change U·G wobble base pairs to stronger canonical G-C base pairs. While U43C alone had a minimal decrease in heat induction, notably, UU4748CC abolished all heat induction. Deletion of the predicted bulged G nucleotide (ΔG44) also results in a notable decrease in heat induction and the combination of U43C/ΔG44 completely abolished heat induction. These mutations demonstrate the importance of the ROSE-like motif of the *P. stuartii oppF* RNA thermometer for the thermoregulation of downstream genes. Interestingly, the U·G wobble base pairs directly downstream of the ROSE-like motif (UU4748), which are also base paired with the SD, are pertinent to the thermometer function.

**Figure 4:**
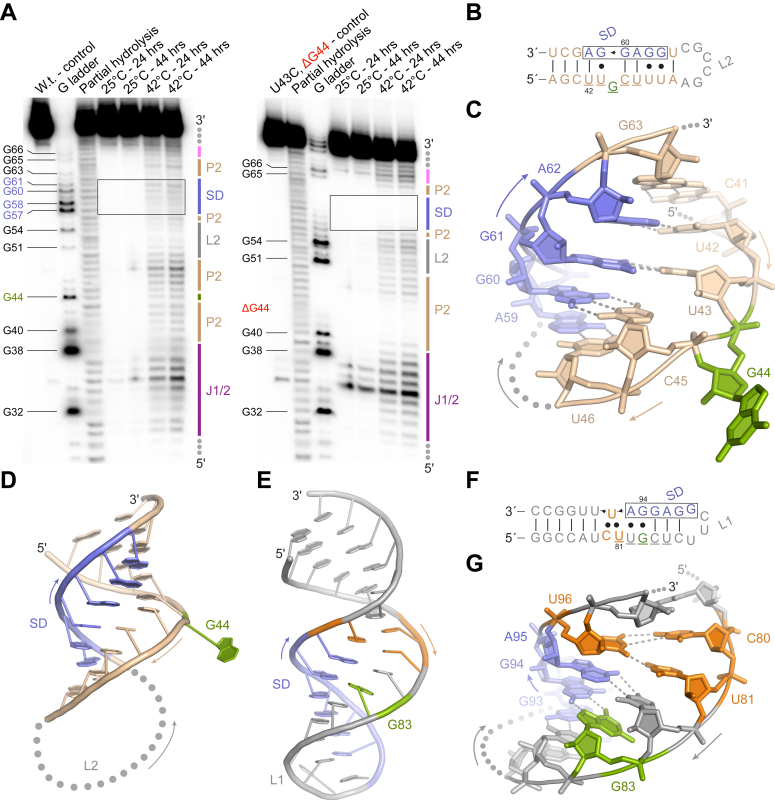
**Structural investigations of the *P. stuartii oppF* ROSE-like RNA thermometer.***A*, in-line probing of wild-type *oppF* (*left*) and U43C/ΔG44 *oppF* mutant (*right*). Shine-Dalgarno region is boxed. Secondary structure features labeled with same colors as Figure 3*B*. *B*, secondary structure representation of the *P. stuartii oppF* P2 ROSE-like RNA thermometer colored as in Figure 3*B*. *C*, cartoon representation detailing the *P. stuartii oppF* ROSE-like motif duplexed with the Shine-Dalgarno element, colored as in Figure 3*B*. *Gray-dashed lines* denote hydrogen bonds. *D* and *E*, cartoon representation comparison of the ROSE-like RNA thermometers P2 from *P. stuartii oppF* (*D*) and the (*E*) previous elucidate *B. japonicum* (PDB: 2GIO). *F*, secondary structure representation of the *B. japonicum* P2 ROSE-like RNA thermometer colored as in (*E*). *G*, cartoon representation detailing the *B. japonicum* ROSE-like motif duplexed with the Shine-Dalgarno element, colored as in (*E*). *Gray-dashed lines* denote hydrogen bonds.

To investigate the effect of conformational changes of the *P. stuartii oppF* RNA thermometer, we directly compared the wild-type *P. stuartii oppF* RNA thermometer to the impaired mutant U43C/ΔG44 using in-line structure probing (Fig. 4*A*). In-line probing provides insights into RNA secondary structure by quantifying the spontaneous cleavage of RNA dependent on local backbone mobility (23). At 25 °C, both the wild-type and the mutant presented limited cleavage, suggesting a stable conformation for both molecules (Fig. 4*A*). However, at 42 °C, the wild-type was considerably less stable than the mutant, particularly in the ROSE-like motif and the SD region, indicating that the RNA is unstructured in these regions. Interestingly, the mutant stabilized the SD to such an extent that it hampered the activity of ribonuclease T1, which cleaves RNA preferentially after guanine residues (Fig. 4*A*). Regions J1/2 and L2 had a similar pattern of hydrolysis for both wild-type and mutant, as expected. Overall, these results confirm the importance of the conserved ROSE-like element for a temperature-dependent conformational change of the *P. stuartii oppF* RNA thermometer.

Next, we investigated if the P2 stem of the *P. stuartii oppF* RNA thermometer can achieve thermoregulation by itself (Fig. 3*C*, Experimental procedures). The P2 stem contains the conserved ROSE-like motif UUGCU (Fig. 2, *C* and *D*). Surprisingly, P2 alone exhibited an increased heat induction at 42 °C of ∼8.4-fold, showing that it is sufficient for complete thermoregulation (Fig. 3*C*).

To further investigate the stereochemical consequences of the conserved ROSE-like motif, we determined the 2.7 Å-resolution X-ray crystal structure of the P2 stem using crystals grown at 21 °C (Figs. 4, *B*–*D*, S3, *A* and *B*, Table S2). The elucidated structure is that of a dimer of *P. stuartii oppF* P2 RNA molecules, in which the two stems are distal from the fortuitous dimer interface. As predicted, G44 is bulged from the stem formed by the ROSE-like motif and the SD. At the crystallization temperature, a wobble (U43·G61) and a canonical (C45–G60) base pair adjacent to the bulged G44 maintains the two RNA strands together. It is known that the nonisostericity (geometric dissimilarity) of the G·U and U·G wobble base pairs impose local structural variation when compared to canonical Watson-Crick base pairs (24, 25). Within the U43·G61 base pair, the glycosidic bond angles of U43 and G61 are 68.8° and 44.5°, respectively, close to the expected ∼70° and ∼40° (24). C45 and G60 also present glycosidic bond angles close to the expected ∼55° for canonical base pairs (C45 = 57.2° and G60 = 57.6°). The U of U·G wobbles present an intrinsic inability to base stack to the succeeding nucleotide (26). The bulged G44 enhances the inability of U43 to stack with the succeeding nucleotide of the helix, C45, likely decreasing the stability of the stem and reducing its melting temperature. This is even more noticeable when considering the distance of the glycosyl carbon atoms C1′ of U43 and C45, measured at 7.6 Å, considerably higher than the average 5.5 Å of the rest of the C1′ atoms of the RNA molecule. In addition, the bulged G44 seems to exacerbate the helical twist angles between the two flanking base pairs, with the preceding U43·G61 being overwound at 40.7° and the succeeding C45–G60 being underwound at 26.7°. The average angle of twist in an A-form RNA is 33°, and G·U and U·G wobble base pairs promote an underwound-overwound pattern of the twisted angles (24). Combined, these structural features may play an important role in the destabilization of the stem at higher temperatures.

Interestingly, our results differ from a previously elucidated NMR structure of a ROSE-like motif–containing stem from *Bradyrhizobium japonicum* (27). In that structure, the natural tetraloop CUUG was mutated into a more stable UUCG tetraloop, and surprisingly, the predicted bulged G83 forms an intramolecular Watson-Crick–Hoogsteen base pair with a guanosine (G94) from the opposite strand of the stem (Fig. 4, *D*–*G*). Another unusual feature in that structure is the interaction involving C80, U81, and U96, where C80 and U81 stack on each other and both interact with the opposite U96 (27) (Fig. 4, *E*–*G*). These architectural distinctions may arise from the difference in nucleotides surrounding the ROSE-like motif and the distance of the motif and the SD to the loop region. Indeed, it suggests that ROSE-like–driven temperature sensing may employ a manifold of RNA conformations.

## Discussion

ABC transporters are ubiquitous in all kingdoms of life and regulate the transport of a wide variety of molecules essential for cell metabolism and survival (2, 28). Specifically in bacteria, ABC transporters are important for adapting to their environment, including a wide variety of hosts. The transport of essential substrates is known to play a major role in the resistance to environmental stresses, such as osmotic and oxidative, which contributes to survival in their hosts (3). Although ABC transporters in bacteria are key in the response to environmental conditions, the interplay between heat stress and ABC transporter expression is not fully understood. Interestingly, ABC transporter genes have been demonstrated to be differentially expressed in bacteria under heat stress, but the mechanism of regulation has remained mysterious (29, 30, 31). However, these studies have focused on the differential expression of ABC transporters in response to temperature at the transcriptome level. Our results suggest that these genes are regulated on the translational level by RNA thermometers.

To date, the majority of characterized RNA thermometers have been discovered upstream of genes encoding heat shock and virulence genes. RNA thermometers have also been reported upstream of non-heat shock and non-virulence-associated genes, such as those found upstream of genes associated with glycerol transport, oxidative stress protection, amino acid biosynthesis, and the ABC transporter gene *oppA* (14, 32). We used our pipeline, Robo-Therm, which uses a combination of a highly customizable motif-driven program and common bioinformatic techniques to discover the widespread occurrence of ROSE-like RNA thermometers upstream of ABC transporters. Our motif-driven bioinformatic approach exemplifies the importance of using a primarily structure-based approach like Robo-Therm in the discovery of new RNA thermometers. Previously, transcriptome probing led to the discovery of a non-ROSE-like RNA thermometer upstream of *oppA* in *Y. pseudotuberculosis* (14). The divergent evolution of RNA thermometer motifs upstream of ABC transporter genes suggests that there are other RNA thermometer sequences with different motifs upstream of ABC transporter genes that have yet to be discovered.

Herein, through structure-guided bioinformatics, we revealed that RNA thermometers upstream of ABC transporter genes are significantly more common in the bacterial kingdom than previously known. We demonstrate temperature-dependent gene regulation by ROSE-like RNA thermometers in the 5′-UTRs of five different ABC transporter genes in diverse bacteria. Our biochemical and structural characterization of the *P. stuartii oppF* RNA thermometer indicates that thermoregulation is achieved post-transcriptionally and is dependent on the ROSE-like motif. This study expands knowledge of the phylogenetic extent of regulation by RNA thermometers of ABC transporter genes, one of the largest gene superfamilies in bacteria.

## Experimental procedures

### RNA motif search in genomic sequences

Bacterial genomic sequences were downloaded from the NIH National Library of Medicine – National Center for Biotechnology Information using the Nucleotide search (https://www.ncbi.nlm.nih.gov/nuccore/). RNArobo (19) was used to perform RNA motif search utilizing the following descriptor:

h1 s1 h2 s2 h2' h1' s3 h3 s4 h4 s5 h4' h3' s6

h1 0:0:2 NNNNNNNN:NNNNNNNN

s1 0:2 N

h2 0:0 NNN:NNN

s2 0 NNNNN

s3 0 NNNNNNN

h3 0:0:2 NCUU:GAGN

s4 0 G

h4 0:0:4 CUNN:NNAG

s5 0:3 NNN

s6 0 ∗∗∗∗∗∗∗∗AUG∗∗∗

Results were manually curated and verified with Basic Local Alignment Search Tool (BLAST) – NIH National Library of Medicine – National Center for Biotechnology Information (https://blast.ncbi.nlm.nih.gov/). Additional results from BLAST were added to the results.

### Phylogenetic tree

Analysis for phylogenetic tree comprised of 41 sequences containing the ROSE-like motif in the P2 stem of predicted ROSE-like thermometers (Table S1). Sequences were aligned *via* muscle alignment and constructed with the maximum likelihood method in MEGA X (33). Branch lengths reflect the amount of genetic change between taxa. Tree was visualized in iTOL (34).

### Plasmid construction

Plasmids were synthesized from VectorBuilder (VectorBuilder Inc, Chicago, IL, USA). The 5′-UTR of thermometer candidates (Table S3) were placed directly upstream of a heat-stable β-galactosidase from *Bacillus stearothermophilus* (35) and driven by a pBAD promoter (pBAD: β-galactosidase). ATG (start codon) in the thermometer sequences replaces the first ATG of *bgaB*. Full vector sequence can be retrieved from the VectorBuilder database using each unique vector ID (https://en.vectorbuilder.com/design/retrieve.html). Vector information is listed below:

VB ID Sequence.

VB230329-1601kpw oppF.

VB220714-1525xxb KY5.

VB230329-1610ysu D3X13.

VB220714-1527fct C3943.

VB230329-1605jyc H7F35.

VB220225-1021dnq agsA.

VB220225-1023wxw gyrA.

NEBuilder HiFi DNA Assembly was used to insert mutation sequences into the same plasmid backbone described above. NEBuilder HiFi DNA Assembly was performed according to the manufacturer's protocol. A previously described vector used to validate the *blyA* thermometer (20) (VectorBuilder ID: VB220225-1020jdm) was used as the backbone for plasmid construction. NEBuilder Assembly Tool 2.0 was used to design fragments. Sequences of mutants for β-galactosidase assay (Table S3) were designed with the following complementary flanking sequences to the VB220225-1020jdm plasmid:

5′- ATACCCGTTTTTTGGGCTAA - Sequences for β-galactosidase assay (Table S3) - AATGTGTTATCCTCAATTTG -3′

### β-Galactosidase assays

*E. coli* DH5α cells carrying *bgaB* plasmids were grown overnight at 25 °C in LB broth plus 100 μg/ml ampicillin. Overnight cultures were diluted in LB broth plus 100 μg/ml ampicillin to an optical density at 600 nm (OD600) of 0.1, and then grown at 25 °C to an OD600 of 0.3 to 0.5. Transcription was induced with 0.01% (w/v) arabinose addition, then cultures were split and incubated at 25, 37, or 42 °C. After 60 min, 500 μl samples were taken, OD600 was measured, and samples were used for β-galactosidase assays as previously described (20, 36, 37) with the following modifications. Three 20 μl samples of culture were added to 80 μl of permeabilization solution (0.8 mg/ml hexadecyltrimethylammonium bromide, 0.4 mg/ml sodium deoxycholate, 100 mM Na_2_HPO_4_, 20 mM KCl, 2 mM MgSO_4_, and 5.4 μl/ml β-mercaptoethanol). After a 30-min incubation at 30 °C, 600 μl of substrate solution (60 mM Na_2_HPO_4_, 40 mM NaH_2_PO_4_, 1 mg/ml o-nitrophenyl-β-D-Galactoside (ONPG), 2.7 μl/ml β-mercaptoethanol) was added. The reactions were incubated at 55 °C for 90 min. The addition of 700 μl of 1 M Na_2_CO_3_ terminated the reactions to be prepared for absorbance readings. Assays were performed in triplicate. Heat induction factor is calculated by dividing expression in Miller Units at 37 or 42 °C by expression at 25 °C.

### Quantitative real time-PCR (qRT-PCR)

Cells were harvested under the same conditions of β-galactosidase assays. Samples for comparative qRT-PCR and β-galactosidase assays were taken from the same cultures post-incubation at 25, 37, or 42 °C. RNA was isolated, treated with DNAse I, and purified using a Directzol RNA Miniprep kit (Zymo). After purification, 150 ng RNA were used for reverse transcription reactions performed using SuperScript IV Reverse Transcriptase kit (Invitrogen) according to the manufacturer’s instructions. cDNA was amplified and detected in the QuantStudio 3 Real-Time PCR System, using the PowerTrack SYBR Green Master Mix (ThermoFisher) and specific primers (Table S3) for *oppF* and *gyrA*. Assays were performed with three biological replicates, each with three technical triplicates. For each gene target, a five-point standard curve was performed. Primer efficiencies calculated by the QuantStudio software: *oppF*: 103.1%, *gyrA*: 101.6%. Relative *bgaB* transcript amounts were calculated using the ΔΔCt method (38), and experimental Ct values were normalized to the non-thermoregulated reference gene *gyrA*.

### In-line structure probing

RNAs were transcribed from PCR templates (purchased from IDT) with T7 RNA polymerase, and purified by denaturing gel electrophoresis (10% polyacrylamide, 29:1 acrylamide: bisacrylamide; 1 × TBE, 8 M urea). After ultraviolet shadowing and excision from gels, RNAs were eluted from the gel into 300 μl of 300 mM KCl and precipitated by adding 700 μl of 100% ethanol at −20 °C. RNAs were resuspended in water. 5′ phosphates were removed by standard protocol utilizing Calf Intestinal Alkaline Phosphatase. Next, RNAs were 5′-^32^P labeled according to standard procedure utilizing T4 polynucleotide kinase and [γ-^32^P]ATP.

The 5′-^32^P end labeled RNAs (10,000 cpm) were incubated for up to 44 h at 25 or 42 °C in buffer containing 140 mM KCl, 20 mM HEPES (pH 8.5), and 1 mM MgCl_2_. Reactions were quenched in a solution of 95% Formamide and 25 mM EDTA. The partially hydrolyzed RNAs were resolved on a 10% denaturing PAGE gel. The gel was exposed to a phosphor image screen (GE Healthcare), and scanned on a GE Typhoon phosphor imager. The sequences in the degradation pattern were assigned by running ribonuclease T1 digestion and alkaline hydrolysis in parallel lanes, as previously reported (23).

### Crystallization and diffraction data collection

RNA (Table S3) was chemically synthesized (Integrated DNA Technologies). RNAs were resuspended in 20 mM MOPS-KOH pH 7.0, 150 mM KCl, and 10 μM EDTA and stored at −20 °C. Prior to use, RNA was heated to 85 °C for 3 min then allowed to cool at 21 °C for 10 min, followed by the addition of MgCl_2_ to 1 mM.

Crystallization was performed by the hanging drop vapor diffusion method. 0.5 μl of RNA solution (250 μM) and 0.5 μl of reservoir solution containing 0.2 M ammonium sulfate, 0.1 M sodium acetate trihydrate pH 4.6, and 30% w/v polyethylene glycol monomethyl ether 2000 were mixed and equilibrated at 21 °C. Cubic-shaped crystals grew to maximum dimensions of 200 × 200 × 200 μm^3^ over 10 to 20 days. Two minutes after the addition of 0.5 ml of cryoprotectant solution (0.1 M ammonium sulfate, 0.05 M sodium acetate trihydrate pH 4.6, 15% w/v polyethylene glycol monomethyl ether 2000, and 20% v/v ethylene glycol) to the drops, crystals were mounted in nylon loops and flash-frozen by plunging into liquid nitrogen. Diffraction data (Table S2) were collected at 100 K using the rotation method at beamline 5.0.1 of the Advanced Light Source (ALS). Datasets were reduced using xia2 (39) with DIALS (40).

### Structure determination and refinement

The structure of *oppF* P2 was solved by molecular replacement using Phaser-MR (41) and a search model consisting of a five-base pair RNA duplex. The solutions were subjected to manual rebuilding in Coot (42) interspersed with rounds of simulated annealing, energy minimization, and individual B-factor refinement in Phenix (43). The mean precision of atomic coordinates was estimated using Phenix. Refinement statistics are summarized in Table S2. Structural figures were prepared with PyMOL (44). The crystallographic asymmetric unit contains a dimer of *oppF* P2 RNA molecules. The structure of the stem is independent of the dimerization and Figure 4*B* depicts a single molecule. Because dimerization interferes with the structure of the L2 loop, it is not depicted in Figure 4, *B* and *C*. The full dimer structure is shown in Fig. S4, *A* and *B*. Analyses of bonds and angles were performed using PyMOL (44) and Web 3DNA 2.0 (45).

## Data availability

The data underlying this article are available in the article and in its online Supporting Information material. Atomic coordinates and structure factor amplitudes have been deposited into the Protein Data Bank (PDB) database under accession code 8VFS.

## Supporting information

This article contains supporting information.

## Conflict of interest

The authors declare that they have no conflicts of interest with the contents of this article.
