# Supplementary material for: RNA thermometers are widespread upstream of ABC transporter genes in bacteria
Source: J Biol Chem. 2024 Jul 9;300(8):107547. doi: 10.1016/j.jbc.2024.107547 (PMC11342760; doi:10.1016/j.jbc.2024.107547)
Supplement: Supporting Information [file mmc1.docx]

RNA thermometers are widespread upstream of ABC transporter genes in bacteria

**SUPPORTING INFORMATION**

**Supporting Figures S1–S3 with legends and Tables S1–S3 with legends**

**Supporting Figure S1**

**
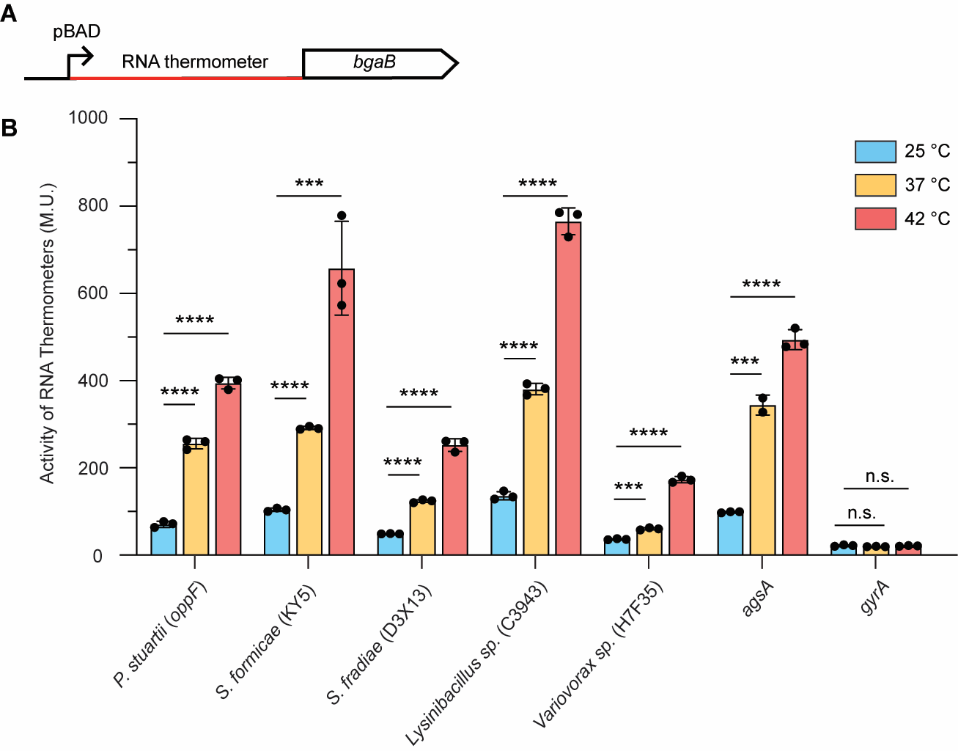
**

**Figure S1. Validation of thermoregulatory activity of RNA thermometers.** (A) Scheme depicting *bgaB* gene fusion with pBAD promoter system used for thermoregulatory activity determination of RNA thermometers based on β-galactosidase assays. (B) Expression in Miller Units (M.U.) of five ROSE-like RNA thermometers at 25, 37, and 42 °C compared to a positive control (*agsA* RNA thermometer) and a negative control (DNA gyrase - *gyrA*). (mean ± s.d., n = 3 biological replicates). For all five candidates, translation was significantly greater at 37 and 42 than at 25 °C with *** (*p* < 0.001), and **** (*p* < 0.0001); Student’s two-tailed *t* test.

**Supporting Figure S2**

**
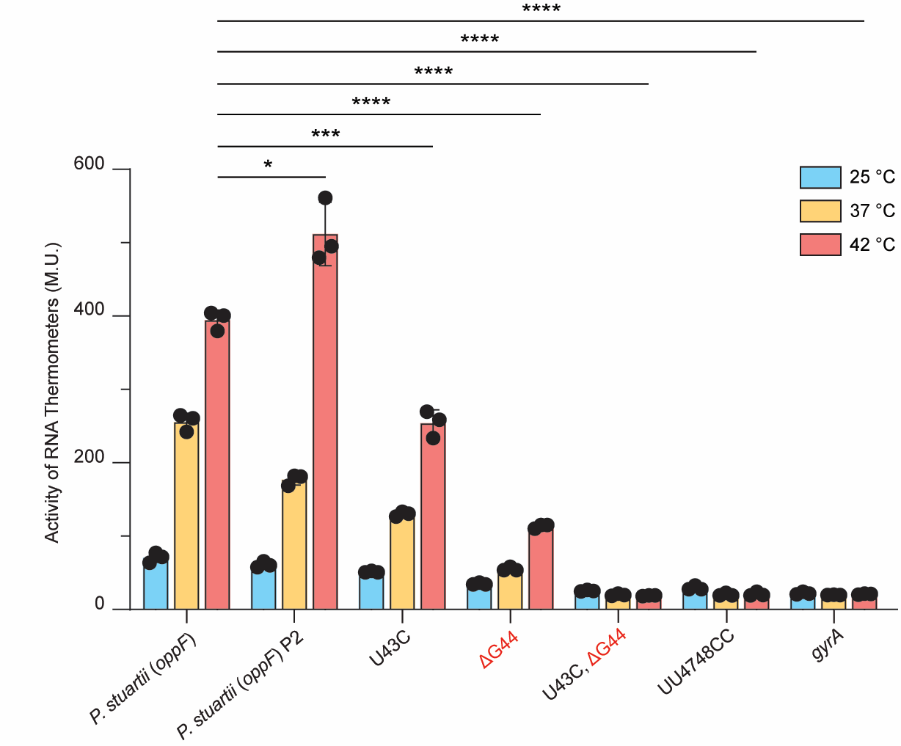
**

**Figure S2. Thermoregulatory activity of mutants.** Expression in Miller Units (M.U.) of *oppF* mutants at 25, 37, and 42 °C compared to *oppF* wild-type and a negative control (DNA gyrase - *gyrA*). (mean ± s.d., n = 3 biological replicates). Translation of the *oppF* wild-type UTR was significantly different from translation of *oppF* mutants at 42 °C with * (*p* < .05), *** (*p* < 0.001), and **** (*p* < 0.0001); Student’s two-tailed *t* test.

**Supporting Figure S3**


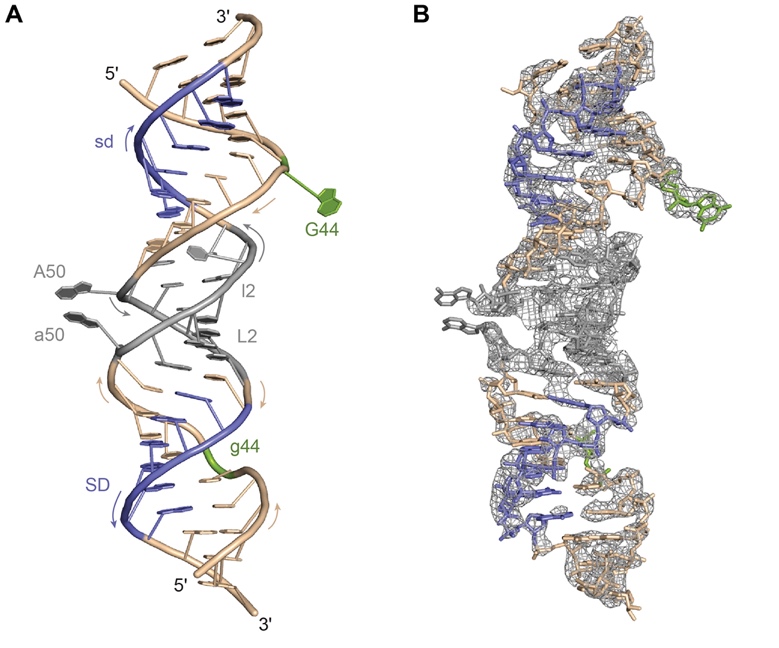


**Figure S3. Dimer structure and electron density map for the structure determined in this study.** (A) Molecule A is labeled with uppercase letters, and molecule B with lowercase letters. Arrows indicate 5′ to 3′ chain direction. (B) Composite simulated annealing-omit 2|*F_o_*| - |*F_c_*| electron density map for the *oppF* P2 RNA dimer contoured at 1.5 *σ* (gray mesh).

**Supporting Table S1. ROSE-like RNA thermometer candidates found upstream of ABC transporter genes in bacteria.**

| **Bacteria** | **Gene encoded protein** | **Sequence of P2 stem containing potential ROSE-like motif, Shine-Dalgarno, and start codon** (5′ to 3′) |
| --- | --- | --- |
| *Anaerolineaceae bacterium* | ABC transporter ATP-binding protein | CTCTTGCTTGCTGTAAGGAGAGTGAACCATG |
| ***Variovorax sp.**** | ABC transporter substrate-binding protein | CTCTTGCTTTCCCAGAAGGAGAAGAACATG |
| *Acidovorax sp.* | ABC-type multidrug transport system, permease component | ATCTTGCTGTTACGCAAGCAGGAGACCTGACATG |
| *Limosilactobacillus reuteri* | ATP-binding cassette domain-containing protein | CTTGCTTTGTTTGAAAGGAGGATGCGTCACGATG |
| *Limosilactobacillus vaginalis* | ATP-binding cassette domain-containing protein | CTTGCTTTGTTTGAAAGGAGGATGCGTCACGATG |
| *Streptomyces alboniger* | BMP family ABC transporter substrate-binding protein | CTTGTGCTTGCTTGACTCAAGGAGTTCGTCTCTATG |
| *Streptomyces alfalfae* | BMP family ABC transporter substrate-binding protein | CTTGTGCTTGCTTGACTCAAGGAGTTCGTCTCTATG |
| *Streptomyces durmitorensis* | BMP family ABC transporter substrate-binding protein | CTTGTGCTTGCTTGTACATAAGGAGTTCCTCTCTATG |
| ***Streptomyces fradiae**** | BMP family ABC transporter substrate-binding protein | CTTGTGCTTGCTTGACTCAAGGAGTTCGTCTCTATG |
| *Streptomyces huasconensis* | BMP family ABC transporter substrate-binding protein | CTTGTGCTTGCTTGACTCAAGGAGTTCGTCTCTATG |
| *Streptomyces kanamyceticus* | BMP family ABC transporter substrate-binding protein | CTTGTGCTTGCTTGTACTAAGGAGTTCGTCTCTATG |
| *Streptomyces venezuelae* | BMP family ABC transporter substrate-binding protein | CTTGTGCTTGCTTGTACTAAGGAGTTCGTCTCTATG |
| *Bradyrhizobium amphicarpaeae* | branched-chain amino acid ABC transporter permease | GGTTGCTGGGCCGCGCCGAGGAGCGCACGGTATG |
| *Bradyrhizobium arachidis* | branched-chain amino acid ABC transporter permease | GATTGCTCGGCCGCGCCGAGGAGCGCACGGTATG |
| *Bradyrhizobium barranii* | branched-chain amino acid ABC transporter permease | GCTTGCTCGGCCGCGCCGAGGAGCGCACCGTATG |
| *Bradyrhizobium betae* | branched-chain amino acid ABC transporter permease | GATTGCTCGGCCGCGCCGAGGAGCGCACGGTATG |
| *Bradyrhizobium commune* | branched-chain amino acid ABC transporter permease | GATTGCTTGGTCGCGCCGAGGAGCGCACGGTATG |
| *Bradyrhizobium daqingense* | branched-chain amino acid ABC transporter permease | GCTTGCTCGGCCGCGCCGAGGAGCGCACCGTATG |
| *Bradyrhizobium japonicum* | branched-chain amino acid ABC transporter permease | GCTTGCTCGGCCGCGCCGAGGAGCGCACCGTATG |
| *Bradyrhizobium sp.* | branched-chain amino acid ABC transporter permease | GCTTGCTCGGCCGCGCCGAGGAGCGCACCGTATG |
| *Bradyrhizobium vignae* | branched-chain amino acid ABC transporter permease | GCTTGCTCGGCCGCGCCGAGGAGCGCACGGTATG |
| *Bradyrhizobium yuanmingense* | branched-chain amino acid ABC transporter permease | GCTTGCTCGGCCGTGCCGAGGAGCGCACCGTATG |
| *Raoultella terrigena* | cysteine/glutathione ABC transporter ATP-binding protein/permease CydC | CCTTGCTGGCCCATCGTCAGGAGGAGATTTAAATG |
| *Escherichia coli* | cysteine/glutathione ABC transporter ATP-binding protein/permease CydC | CCTTGCTGGCCCATCGTCAGGAGGAGATTTAAATG |
| *Escherichia fergusonii* | cysteine/glutathione ABC transporter ATP-binding protein/permease CydC | CCTTGCTGGCCCATCGTCAGGAGGAGATTTAAATG |
| *Shigella sp.* | cysteine/glutathione ABC transporter ATP-binding protein/permease CydC | CATTGCTGGCCCATCGTCAGGAGGAGATTTAAATG |
| ***Lysinibacillus sp.**** | methionine ABC transporter ATP-binding protein | GCAGATTCTTGCTTTTTAGAAAGGAGACATCCATATG |
| *Lysinibacillus pakistanensis* | methionine ABC transporter ATP-binding protein | GCAGATTCTTGCTTTTTAGAAAGGAGACATCCAGATG |
| ***Streptomyces formicae**** | nucleoside ABC transporter, periplasmic nucleoside-binding protein | GCTTGTGCTTGCTTGTACTAAGGAGTTCGTCTCTATG |
| *Buttiauxella ferragutiae* | oligopeptide ABC transporter ATP-binding protein OppF | GCTTGCTTTAAGCCAGTGGAGGAGTTGGTATG |
| *Buttiauxella sp.* | oligopeptide ABC transporter ATP-binding protein OppF | GCTTGCTTTAAGCCAGTGGAGGAGTTGGTATG |
| *Providencia alcalifaciens* | oligopeptide ABC transporter ATP-binding protein OppF | GCTTGCTTTAAGCCAGTGGAGGAGTTGGTATG |
| *Providencia manganoxydans* | oligopeptide ABC transporter ATP-binding protein OppF | GCTTGCTTTAAACCAGTGGAGGAGTTGCTATG |
| ***Providencia stuartii**** | oligopeptide ABC transporter ATP-binding protein OppF | GCTTGCTTTAAGCCGCTGGAGGAGCTGGTATG |
| *Providencia thailandensis* | oligopeptide ABC transporter ATP-binding protein OppF | GCTTGCTTTAAGCCGGTGGAGGAGCTGGTATG |
| *Providencia vermicola* | oligopeptide ABC transporter ATP-binding protein OppF | GCTTGCTTTAAGCCAGTGGAGGAGTTGGTATG |
| *Shigella dysenteriae* | oligopeptide ABC transporter ATP-binding protein OppF | GCTTGCTTTAAACCGGTGGAGGAACTGTTATG |
| *Shigella flexneri* | oligopeptide ABC transporter ATP-binding protein OppF | GCTTGCTTTAAACCGGTGGAGGAACTGTTATG |
| *Winslowiella toletana* | oligopeptide ABC transporter ATP-binding protein OppF | GCTTGCTTTAAGCCAGTGGAGGAGTTGGTATG |
| *Yersinia entomophaga* | oligopeptide ABC transporter ATP-binding protein OppF | GCTTGCTTTAAGCCAGTGGGGGAATTGGTATG |
| *Antarctobacter heliothermus* | peptide ABC transporter ATP-binding protein | ACTTGCTGAGCGACGGTCTTAGGAGTGCCATG |

***** Five RNA thermometer candidates tested and confirmed as shown in **Fig. 2**.

**Supporting Table S2. Crystallographic statistics.**

|  | *oppF* P2  RNA thermometer |
| --- | --- |
| **Data collection*^†^** |  |
| Beamline | ALS 5.01 |
| Wavelength (Å) | 0.9774 |
| Space group | *P* 43 2_1_ 2 |
| Cell dimensions |  |
| *a*, *b*, *c* (Å) | 63.60 63.60 86.15 |
| *α*, *β*, *γ* (°) | 90 90 90 |
| Resolution (Å) | 51.17 - 2.7 |
| *R*_merge_ | 0.094 (0.712) |
| *CC*_1/2_ | 1 (0.995) |
| *<I>*/<σ(*I)*> | 18.53 (2.22) |
| Completeness (%) | 97.63 (97.16) |
| Redundancy | 24.0 (25.5) |
|  |  |
| **Refinement** |  |
| Resolution (Å) | 51.17 - 2.7 (2.97 - 2.7) |
| No. reflections | 5108 (1231) |
| *R*_work_ / *R*_free_ | 0.211 / 0.239 |
| No. atoms |  |
| RNA | 1137 |
| Ligand/ion | 0 |
| Water | 4 |
| *B*-factors (Å^2^) |  |
| RNA | 70.62 |
| Ligand/ion | - |
| Water | 57.12 |
| R.m.s. deviations |  |
| Bond lengths (Å) | 0.005 |
| Bond angles (°) | 1.06 |
| Mean precision (Å) | 0.41 |
| PDB ID | 8VFS |

*One crystal was used for each dataset. ^†^Values in parentheses are for highest resolution shell.

**Supporting Table S3. Sequences of oligonucleotides used in this study.**

| **Oligonucleotide** | **Description** | **Sequence** (5′ to 3′) |
| --- | --- | --- |
| *oppF ^a^* | Sequence for β-galactosidase assay and in-line probing | GAGCCTGAGCTGAATGCATTTTCAGCGGGTCGATTAAGAGCTTGCTTTAAGCCGCTGGAGGAGCTGGTATG |
| *oppF* – P2*^a^* | Sequence for β-galactosidase assay | GTCGATTAAGAGCTTGCTTTAAGCCGCTGGAGGAGCTGGTATG |
| *KY5* *^a^* | Sequence for β-galactosidase assay | CGGACCGGACAGAAGGGTTCTTATGTCAGGTCTACGCGCGTTACGCTGCGGCGAAATCAGCGCCAGGTGAGGCGCTCGTACGTGCTTGTGCTTGCTTGTACTAAGGAGTTCGTCTCTATG |
| *D3X13* *^a^* | Sequence for β-galactosidase assay | GCCAGGTGAGGCGCTCGTACGTTCTTGTGCTTGCTTGACTCAAGGAGTTCGTCTCTATG |
| *C3943* *^a^* | Sequence for β-galactosidase assay | TAGTGAAACTTTAATCAGCAGGGATTTTCCACAGATTATTAGTTTAAATGCAGGATAAAATAGAGAATCTGCGAGACTCCTCTGGGAAAGCGAGCAGATTCTTGCTTTTTAGAAAGGAGACATCCATATG |
| *H7F35* *^a^* | Sequence for β-galactosidase assay | AAGGTGCCGCTCTTGCTTTCCCAGAAGGAGAAGAACATG |
| ∆G44 *^a^* | Sequence for β-galactosidase assay | GAGCCTGAGCTGAATGCATTTTCAGCGGGTCGATTAAGAGCTTCTTTAAGCCGCTGGAGGAGCTGGTATG |
| U43C *^a^* | Sequence for β-galactosidase assay | GAGCCTGAGCTGAATGCATTTTCAGCGGGTCGATTAAGAGCT**C**GCTTTAAGCCGCTGGAGGAGCTGGTATG |
| ∆G44/ U43C *^a^* | Sequence for β-galactosidase assay and in-line probing | GAGCCTGAGCTGAATGCATTTTCAGCGGGTCGATTAAGAGCT**C**CTTTAAGCCGCTGGAGGAGCTGGTATG |
| UU4748CC *^a^* | Sequence for β-galactosidase assay | GAGCCTGAGCTGAATGCATTTTCAGCGGGTCGATTAAGAGCTTGCT**CC**AAGCCGCTGGAGGAGCTGGTATG |
| *agsA ^a^* | Sequence for β-galactosidase assay | TGTAGTCAGTGGACAAGCAATGCTTGCCTTGATGTTGAACTTTTGAATAGTGATTCAGGAGG TTAATGATG |
| *gyrA ^a^* | Sequence for β-galactosidase assay | ACCTCAAACTGCGCGGCTGTGTTATAATTTGCGACCTTTGAATCCGGGATACAGTAGAGGGAT AGCGGTTAGATG |
| *gyrA* fwd primer | qPCR primer | GTCAGGGTAACTTCGGTTCTATC |
| *gyrA* rvs primer | qPCR primer | GAGATCGGCCATCAGTTCAT |
| *bgaB* fwd primer | qPCR primer | CCGTGGTGCAGATGGTATTAT |
| *bgaB* rvs primer | qPCR primer | CTCTTGCCCTAACTGTGTAACT |
| *oppF* – P2 only | RNA sequence for crystallization | AGCUUGCUUUAAGCCGCUGGAGGAGCU |

*^a^* underlined ATG is the start codon for *bgaB*
